# Success Rate of MTA Pulpotomy on Vital Pulp of Primary Molars: A 3-Year Observational Study

**DOI:** 10.5005/jp-journals-10005-1368

**Published:** 2016-09-27

**Authors:** Brinda Godhi, Rishi Tyagi

**Affiliations:** 1 Reader, Department of Pedodontics, JSS Dental College and Hospital Sri Jagadguru Sri Shivarathreeshwara University, Mysuru Karnataka, India; 2 Associate Professor, Department of Pedodontics, UCMS and GTB Hospital, New Delhi, India

**Keywords:** Mineral trioxide aggregate, Primary molars, Pulpotomy.

## Abstract

**Introduction:**

Vital pulp therapy is a major contributor in the preservation of primary dentition after caries affliction. Introduction of mineral trioxide aggregate (MTA) has revolutionized such treatment.

**Aim:**

The aim of our study was to evaluate and correlate the effects of MTA clinically and radiographically on pulpotomized primary molars till their exfoliation or extraction followed by histological evaluation.

**Study design:**

This is an observational study.

**Materials and methods:**

A total of 25 teeth were selected from 5- to 8-year-old children requiring pulp therapy on the basis of inclusion and exclusion criterion. The teeth were treated by conventional pulpotomy technique under aseptic conditions using MTA and were immediately restored with stainless steel crown. The teeth were assessed postoperatively till 36 months. The exfoliated or extracted teeth were examined histologically.

**Results:**

The pulpotomized teeth were vital with no adverse clinical findings during the observation period. After 3 months, one tooth showed internal resorption, but the same was not observed after 12 months. Pulp canal obliteration was seen in three cases. At the end of the study, five teeth were exfoliated and one tooth was extracted for maintaining arch symmetry. The histological examination of extracted tooth revealed the presence of healthy pulp and the area of true calcification. Remaining exfoliated teeth presented dentin bridge formation.

**Statistics:**

Frequencies and percentages were used for descriptive statistics. Fisher’s exact tests were used to see the difference between clinical and radiological findings. The probability value was fixed at 5% level of significance.

**Conclusion:**

The response of pulp in primary teeth to MTA was favorable in all cases from clinical and radiographic perspective, and histological evaluation confirmed the observation.

**How to cite this article:**

Godhi B, Tyagi R. Success Rate of MTA Pulpotomy on Vital Pulp of Primary Molars: A 3-Year Observational Study. Int J Clin Pediatr Dent 2016;9(3):222-227.

## INTRODUCTION

Pulpotomy is still the most common treatment for cari-ously exposed pulps in symptom-free primary molars. The aim of this treatment was to preserve the vitality of radicular pulp, avoiding pain and swelling, and ultimately to retain the tooth, preserving arch integrity. Several materials have been used as popular pulpotomy medicaments. Formocresol is one such pulpotomy medicament that has been in use for the past 70 years.^1^ Even with acceptable clinical and radiographic results, concerns have been raised about formocresol, because of its association with systemic toxicity and carcinogenic potential in humans, and its safety in children has been questioned.^2-4^ Alternatives have been proposed to maintain the radicular pulp’s vitality. These include electrosurgery,^5,6^ laser,^7^ glutaraldehyde,^8,9^ ferric sulfate,^10^ bone morphogenetic protein,^11^ and osteogenic protein.^12^

Pulpotomy has been broadly classified as devitalization, preservation, and regeneration of the remaining pulp tissue.^13^ One material that has shown immense potential for regeneration is mineral trioxide aggregate (MTA). It is a biocompatible and bioinductive material that has been investigated for endodontic applications since the early 1990s. Mineral trioxide aggregate was first described in the dental scientific literature in 1993^14^ and was given approval for endodontic use by the US Food and Drug Administration in 1998.^15^

Mineral trioxide aggregate pulpotomy has been studied clinically and radiologically extensively and compared with other pulpotomy medicaments, and it has been concluded that MTA can be an acceptable alternative. There are few studies in the literature where the response of human pulp to MTA has been histo-logically studied.^16-18^ Hence, this study was conducted to evaluate and correlate the effects of MTA clinically and radiographically on pulpotomized primary molars till their exfoliation or extraction followed by histological evaluation.

## MATERIALS AND METHODS

Ethical clearance to conduct the study was obtained from the Institutional Review Board. The procedure, possible discomfort, and benefits were explained to parents of the children involved, and written consent was obtained.

A total of 25 maxillary and mandibular primary molar teeth were selected from 19 normal, healthy, and cooperative children aged between 5 and 8 years, requiring pulp therapy from the patients attending the Department of Pedodontics and Preventive Dentistry, ITS Center for Dental Studies and Research, Ghaziabad, Uttar Pradesh, India. Intraexaminer agreement was done using kappa coefficients. The agreement was found to be 100%.

The criteria for selection of teeth to be included in the study are given below.

### Inclusion Criteria

- Symptomless primary molars with a deep carious lesion.
- Exposure of vital pulp due to dental caries.
- No clinical and radiographic evidence of inflamed pulp, pulp degeneration, such as excessive bleeding from root canal, swelling or sinus tract, internal resorption, interradicular bone destruction, no peri-apical bone destruction, and no radiolucency in furcation area.
- Pulp shall be uninflamed and no pain on percussion clinically.
- Teeth should be restorable after completion of the procedure.

### Exclusion Criteria

- Excessive bleeding during pulp amputation.
- Nonvital teeth.

Primary molars were treated by conventional pulp-otomy technique using local anesthesia and isolating the teeth with rubber dam. After caries removal with round bur, coronal access was obtained using a #330 high-speed bur with water spray to expose the pulp chamber. The deroofing of pulp chamber was done by connecting the pulp horns by a nonend-cutting bur. The coronal pulp was excised until root canal orifices, with no tags remaining on the pulpal floor using Hu-Friedy sharp spoon excavator. After the pulp was amputated, the pulp chamber was irrigated with saline to wash away dentin debris. Following irrigation, sterile, saline-wetted cotton pellets were applied for 5 minutes on the amputated pulp stumps to achieve hemostasis. After the standardized technique, pulp stumps were covered with MTA paste that is obtained by mixing MTA powder with distilled water provided by manufacturer in 3:1 (powder-liquid) ratio.

Then, the mixture was compressed against the exposure site with a moist cotton pellet. Wet cotton pellet was placed in pulp chamber, and cavity was covered with intermediate restorative material (IRM). In the second session 1 day after, patients were recalled and cotton pellet was removed. Cavity was restored with IRM, and intra-oral periapical radiographs were taken. Within 1 week, the tooth was restored with a preformed stainless steel crown.

After 1, 3, 6, 12, 24, and 36 months, the children were recalled for clinical and radiographic examination postoperatively. The children were examined clinically, for the presence of signs and symptoms, such as pain, swelling, and sinus/fistula, and radiographically for periapical changes, furcation radiolucency, and internal resorption.

The treatment was regarded as a failure when one or more of the above-mentioned signs and symptoms were present, but pulp canal obliteration was not regarded as a failure. During the follow-up, if the treated teeth exfoliate or extraction was done for balancing, the arch will be subjected to histological examination and it will be taken as success of the treatment.

### Histologic Study

Exfoliated teeth were kept in 10% buffered formalin solution for minimum 24 hours and were decalcified in 5% nitric acid for 3 weeks. Six-micrometer-thick sections were cut with a microtome in buccolingual direction and stained with hematoxylin and eosin and viewed under light microscope. The sections were evaluated by the pathologist according to the criteria described in Table 1.

The frequencies and percentages were used for descriptive statistics. Fisher’s exact tests were used to see the difference between clinical and radiological findings. The probability value was fixed at 5% level of significance. Data were statistically analyzed by Statistical Package for the Social Sciences software version 17.

**Table 1:** **Table 1:** Sections graded - modified scoring system adapted from Stanley (1-3) (Kot et al 1995)^19^

1 Dentin bridge		0 – No presence of bridge formation	
formation		1 – Bridge formation <25%	
		2 – 25% bridge formation <50%	
		3 – 50% bridge formation <75%	
		4 – Bridge formation 75%	
2 Quality of dentin		0 – No tubules present	
formation in the		1 – Irregular pattern of tubules	
dentin		2 – Regular pattern of tubules	
3 Pulpal inflammation		0 – No inflammation	
		1 – Mild inflammation present	
		2 – Moderate inflammation	
		3 – Severe inflammation	

## RESULTS

Out of 25 selected teeth, 6 were first primary molars and 19 were second primary molars. The age of the subjects ranged from 5 to 8 years with mean (±standard deviation) age (years) 6.44 (± 1.12).

**Figs 1A to F:**
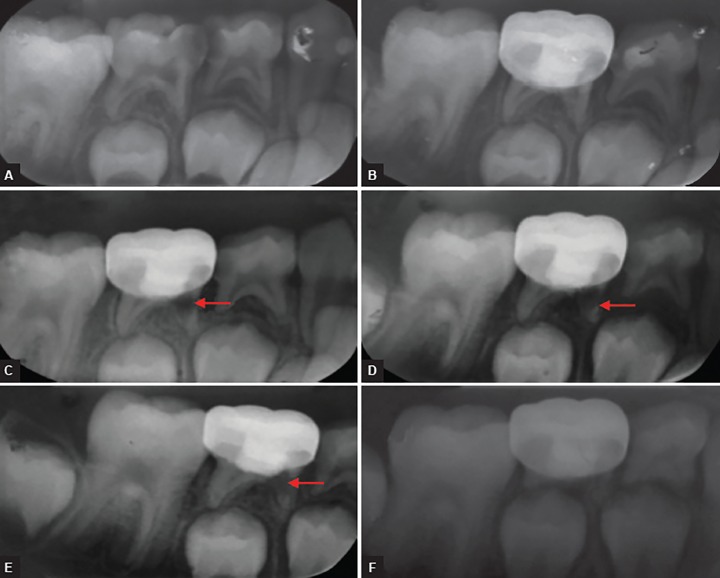
Preoperative and postoperative radiograph of mandibular second primary molar treated with MTA pulpotomy revealed internal resorption; (A) Preoperative radiograph; (B) postoperative radiograph taken immediately after MTA pulpotomy and restored with stainless steel crown; (C) radiograph taken 3 months postoperatively showing feature of failure in pulpotomy; (D) radiograph taken 6 months postoperatively showing feature of failure in pulpotomy; (E) radiograph taken 24 months postoperatively showing internal resorption found to be arrested, and pulp tissue was replaced by calcified tissue; and (F) radiograph taken 36 months postoperatively showing normal physiologic resorption

The follow-up evaluation revealed 100% success for clinical signs and symptoms for all 25 teeth at 1, 3, 6, 12, 24, and 36 months when observed by the same observer. The radio-graphic evaluation at 3 months revealed internal resorp-tion with one tooth, but the same was not observed after 12 months; during the follow-up period of 24 and 36 months, internal resorption was found to be arrested and pulp tissue was replaced by calcified tissue, with tooth being clinically asymptomatic (Figs 1A to F). Pulp canal obliteration was found in the three cases and was not considered as failure. Out of 25 teeth, treated 24 teeth did not show any radiographic changes, giving 96% success at the end of 12 months, and it increased to 100% success at the end of 36 months because one tooth that showed internal resorption at the end of 12 months was found to be arrested and pulp tissue was replaced by calcified tissue. So this tooth was not considered as failure. There were no significant differences after 1, 3, 6, 12, 24, and 36 months’ evaluation periods for clinical and radiographic success (Table 2).

**Table 2:** **Table 2:** Success rate of clinical and radiological outcome

*Follow-up* *(months)*		*Clinical* *succes*		*Radiological* *succes*		*p-value*	
3		25 (100%)		24 (96%)		1.000 NS	
6		25 (100%)		24 (96%)		1.000 NS	
12		25 (100%)		24 (96%)		1.000 NS	
24		25 (100%)		25 (100%)		–	
36		25 (100%)		25 (100%)		–	

NS: Nonsignificant

**Figs 2A and B:**
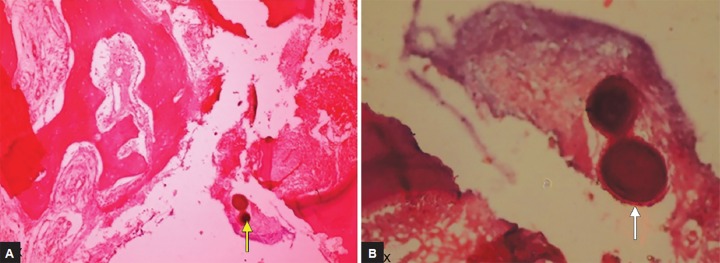
Photomicrograph of a section of tooth treated with MTA showing globular calcified mass

**Figs 3A and B:**
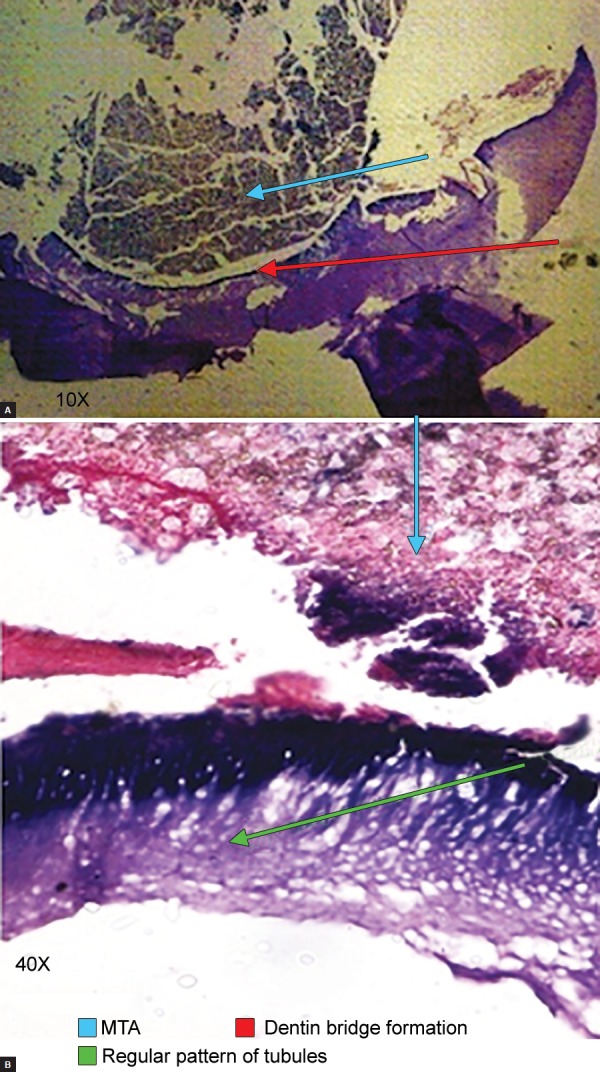
Photomicrograph of a section of tooth treated with MTA showing dentin bridge formation and regular pattern of tubules

At the end of the study, six (24%) teeth were available for histological examination: Five teeth were exfoliated, and one tooth was extracted for maintaining arch symmetry. The histological examination of extracted tooth previously that showed internal resorption revealed the presence of healthy pulp and the area of true calcification (Figs 2A and B). Remaining exfoliated teeth presented dentin bridge formation >25% but <50% with five teeth showing regular pattern of tubules (Figs 3A and B) and one tooth with irregular pattern.

## DISCUSSION

In this study, clinically, the success rate was 100% at all observation periods. The results are in agreement with the results of various previous studies.^20-25^ The clinical success was attributable to proper case selection, high aseptic standards, proper technique protocol, appropriate use of medicament, excellent sealing ability of the material, biocompatibility, alkalinity, and ability to regenerate the hard tissues.^26^

Radiographically, one tooth showed internal resorp-tion at 3 months interval, which did not progress to perforate the root any further over a period of next 9 months. Similar findings were seen at a duration of 25 to 38 months in two cases in a study by Holan et al^27^ and at a duration of 12 months by Jabbarifar et al^28^ respectively.

Initially, it was taken as failure, but during the follow-up at 24 months, there was arrest of progression of internal resorption process with replacement by calcified material. This has been reported earlier in a study by Holan et al.^27^ Smith et al^29^ and Papagiannoulis^30^ have reported arrest of progression of internal resorption with ferric sulfate pulpotomy. Internal resorption may result from overstimulation of primary pulp by the highly alkaline calcium hydroxide. This highly alkaline-induced overstimulation could cause metaplasia within the pulp tissue, leading to the formation of odontoblasts. Although MTA does not contain calcium hydroxide, calcium oxide is formed after MTA hardening, which can react with tissue fluids to produce calcium hydroxide.^31^ According to Seux et al,^32^ after contact with pulp tissue, MTA presents some structures that are similar to calcite crystals found in calcium hydroxide. They attract fibro-nectin, i.e., generally responsible for cellular adhesion and differentiation, as do calcium hydroxide. Therefore, we believe that the mechanism of action of MTA is similar to that of calcium hydroxide.^33^

The aim of pulpotomy was to retain a symptom-free, functional primary tooth until it reaches the age of its physiologic exfoliation. This definition allows the inclusion of any condition and process that is symptom-free and does not impair the tooth’s function. Internal resorption fits this definition, as long as it is not associated with external inflammatory root resorption. Internal resorption can, therefore, be left for follow-up, expecting the arrest of the process and the development of calcific metamorphosis.^27^ It could also be more likely the result of undiagnosed chronic inflammation existing in radicular pulp prior to pulpotomy.^34^

The histological examination revealed the presence of healthy pulp and the area of true calcification in one tooth and dentin bridge formation, i.e., >25% but <50% with regular pattern of dental tubules in remaining five teeth. Similar findings were seen in study on maxillary third molars by Aeinehchi et al,^16^ Iwamoto et al,^35^ Caicedo et al^18^ on primary teeth. Chacko and Kurikose,^17^ in a study on first premolars pulp capping with MTA, have shown homogeneous dentin bridge formation at 4 and 8 weeks. Chueh and Chiang^36^ concluded that, when caries and bacterial contamination is removed from the dentin-pulp complex, the inflamed but vital pulp of a permanent tooth may have a chance to return to a healthy, functional status after MTA pulpotomy.

In this observational study (36 months), histological response showed healthy pulp, dentin bridge formation, and areas of true calcifications under MTA.

## CONCLUSION

Mineral trioxide aggregate on amputated pulpal tissue seems to suggest that the material preserves the pulp tissue and promotes the regeneration of hard tissues. We were fortunate enough to follow-up for 3 years, and treated teeth that exfoliated eventually have been correlated histologically. Pulp remained healthy after 3 years of application of MTA on pulpotomized primary molars. The whole aim of pulpotomy was maintaining integrity of the pulp. Mineral trioxide aggregate has maintained it. Mineral trioxide aggregate has a biological potential for healing and repair. So it can be recommended as a replacement for pulpotomy procedures.
